# Workload is associated with the occurrence of non-contact injuries in professional male soccer players: A pilot study

**DOI:** 10.3389/fpsyg.2022.925722

**Published:** 2022-08-04

**Authors:** Hadi Nobari, Sara Mahmoudzadeh Khalili, Angel Denche Zamorano, Thomas G. Bowman, Urs Granacher

**Affiliations:** ^1^Department of Exercise Physiology, Faculty of Educational Sciences and Psychology, University of Mohaghegh Ardabili, Ardabil, Iran; ^2^Department of Motor Performance, Faculty of Physical Education and Mountain Sports, Transilvania University of Braşov, Braşov, Romania; ^3^Sports Scientist, Sepahan Football Club, Isfahan, Iran; ^4^Faculty of Sport Sciences, University of Extremadura, Cáceres, Spain; ^5^Faculty of Sports Science, University of Isfahan, Isfahan, Iran; ^6^Department of Health and Sport Rehabilitation, Faculty of Sport Sciences and Health, Shahid Beheshti University, Tehran, Iran; ^7^Department of Athletic Training, College of Health Sciences, University of Lynchburg, Lynchburg, VA, United States; ^8^Division of Training and Movement Sciences, University of Potsdam, Potsdam, Germany

**Keywords:** ACWR, external load, football, prevention, performance, injury risk

## Abstract

Injuries in professional soccer are a significant concern for teams, and they are caused amongst others by high training load. This cohort study describes the relationship between workload parameters and the occurrence of non-contact injuries, during weeks with high and low workload in professional soccer players throughout the season. Twenty-one professional soccer players aged 28.3 ± 3.9 yrs. who competed in the Iranian Persian Gulf Pro League participated in this 48-week study. The external load was monitored using global positioning system (GPS, GPSPORTS Systems Pty Ltd) and the type of injury was documented daily by the team's medical staff. Odds ratio (OR) and relative risk (RR) were calculated for non-contact injuries for high- and low-load weeks according to acute (AW), chronic (CW), acute to chronic workload ratio (ACWR), and AW variation (Δ-Acute) values. By using Poisson distribution, the interval between previous and new injuries were estimated. Overall, 12 non-contact injuries occurred during high load and 9 during low load weeks. Based on the variables ACWR and Δ-AW, there was a significantly increased risk of sustaining non-contact injuries (*p* < 0.05) during high-load weeks for ACWR (OR: 4.67), and Δ-AW (OR: 4.07). Finally, the expected time between injuries was significantly shorter in high load weeks for ACWR [1.25 vs. 3.33, rate ratio time (RRT)] and Δ-AW (1.33 vs. 3.45, RRT) respectively, compared to low load weeks. The risk of sustaining injuries was significantly larger during high workload weeks for ACWR, and Δ-AW compared with low workload weeks. The observed high OR in high load weeks indicate that there is a significant relationship between workload and occurrence of non-contact injuries. The predicted time to new injuries is shorter in high load weeks compared to low load weeks. Therefore, the frequency of injuries is higher during high load weeks for ACWR and Δ-AW. ACWR and Δ-AW appear to be good indicators for estimating the injury risk, and the time interval between injuries.

## Introduction

Soccer is a high-intensity intermittent team sport that involves vigorously high and low exercises, and requires a significant level of endurance (Draganidis et al., 2013). Mean injury incidence, considering matches and training sessions across seven UEFA clubs during seven seasons, was reported as 50 injuries per season in a team (*N* = 25) which equals two injuries per player and season (Ekstrand et al., 2011b). Non-contact injuries like anterior cruciate ligament injuries, occurred most often due to the sudden deceleration and repeatedly performed pivoting maneuvers (Boden et al., 2000). According to the Anderson et al. study on the mechanisms of soccer injuries, 23% of injuries are non-contact type (Andersen et al., 2004). Therefore, injuries in professional soccer are a significant concern for teams, and injury prevention in elite soccer should be considered important. Most injuries are caused by high training load. In the meantime, external training load which consists of various training variables such as body load (BL), acceleration, speed, metabolic power, etc. can be used as a monitoring tool to prevent injuries. Therefore, different workload parameters obtained from training load have been used to detect injury risk in different activities (Jones et al., 2017).

Training load is usually classified into measures of external and internal load. External load refers to all movements of the players, and can be assessed by some micro electromechanical devices such as global positioning systems (GPS), local positioning systems, and inertial measurement units (Gabbett, 2016); while internal load refers to the athletes' biological responses to an external load, such as heart rate and ratings of perceived exertion (Bourdon et al., 2017). GPS is the most used tracking tool to collect external workloads during training in team sports (Akenhead and Nassis, 2016), and it has been shown that GPS is a valid and reliable tool to monitor training (Nikolaidis et al., 2018). Some studies on the workload and related injuries among professional soccer players have confirmed the importance of monitoring the workload of players to prevent injury (Jaspers et al., 2017; Malone et al., 2017). Typically, acute (AW), chronic (CW), and AW/CW workload ratio (ACWR) are good indicators to reflect the relationship between fatigue (related to AW) and fitness (related to CW) of the players, which have been associated with non-contact injury (Murray et al., 2017a; Weiss et al., 2017). Players with a high CW were more resistant to injury compared to players who had a low CW (Hulin et al., 2016a). Overall, when the ACWR is within the range of 0.8-1.3 arbitrary unit (A.U.), the injury probability is low (“sweet spot”), and when this ratio exceeds 1.5 A.U., the injury probability doubles (Soligard et al., 2016). Malone et al. (2016b) reported that there is an association between high ACWR >2.0 A.U. and increased probability of injury within team sport athletes. In cricket, using ACWR, it has been demonstrated that higher CW protect against injury (Hulin et al., 2014). Impellizzeri et al. (2020a) concluded that no evidence suggests the use of ACWR in managing training loads to reduce injury risk. However, Gabbett (2016) stated that excessive and rapid increases in training loads are probably responsible for most non-contact injuries. In 2016, the International Olympic Committee published a consensus statement which suggests the use of the ACWR approach for injury prevention (Schwellnus et al., 2016). The biggest concern of soccer players is time loss due to injury (Ekstrand et al., 2011a) and imposing heavy costs on the soccer staff (Lehmann and Schulze, 2008). Therefore, monitoring players' workload may help to reduce the risk of sustaining injuries, time off from competitions, and the costs related with injuries.

Although, recently, many studies have investigated the effect of workload parameters, especially ACWR, on different types of injuries in soccer players, what differentiates this study from others is its target population. It can be said with confidence that this study is the first of its kind in the level of Asian leagues. Therefore, considering the incidence of non-contact injuries in soccer players, the importance of workload monitoring to prevent non-contact injuries in professional soccer players, and mixed ideas in the literature about these two factors, this study aimed to examine the role of the incidence rate for non-contact injuries, during high vs. low load weeks across a full soccer season using odds ratio (OR) and relative risk (RR). Also, an investigation into the relationship between AW, CW, ACWR, and AW variation (Δ-Acute) with non-contact injury incidence rate in professional players throughout a full soccer season and ultimately, the estimation of the predicted time between old injuries to new injury using Poisson distribution were conducted. With reference to the relevant literature (Malone et al., 2016b), we hypothesized that high training load per week will be associated with higher non-contact injury (OR and RR).

## Materials and methods

### Participants

Twenty-one professional soccer players aged 28.3 ± 3.9 yrs from the Iranian Persian Gulf Pro League volunteered to participate in this study for a full season. All team players were monitored during training sessions. However, goalkeepers were excluded from study participation. The criterion was participants' information, which was entered into the analysis based on their attendance of at least three training sessions per week. The criterion for excluding participants' data from the analysis were that the data had not been available for at least two weeks or that they had not participated in the training for at least two weeks (Nobari et al., 2021). The coaching staff of the soccer team, after obtaining permission from the relevant authorities and the head coach of the club, designed and programmed the soccer training. Before commencing the study, it also received the approval of the research ethics committee from the University of Mohaghegh Ardabili and University of Isfahan. All players were informed of the purpose of the study before completing the informed consent. All stages of this study were carried out based on the ethical principles in the Helsinki Declaration.

### Study design

This longitudinal study was conducted during a full season (48 weeks) of the Persian Gulf Pro League and knockout tournament. GPS (GPSPORTS systems Pty Ltd, Model: SPI High-Performance Unit (HPU), Canberra, Australia) was used for monitoring the external load at each training and match session during the whole season. All non-contact injuries were recorded during the season. Overall, 7-weeks congested (i.e., two or more matches within 7-days), 30-weeks non-congested, 44 matches, 200 training sessions, and 14,126.8 min of time played and sessions were held. The training sessions and matches took place on natural grass pitches. All workload parameters including AW, CW, ACWR, and Δ-Acute were calculated. Afterwards, each variable was divided into two levels, high and low load, and subsequently, the relationship between the variables was measured.

### Procedures

#### External load

##### GPS receiver specifications

GPSPORTS systems Pty Ltd, recorded all players' activities in training sessions and matches. The GPS-based tracking systems for professional athletes, model SPI HPU features included: 15 Hz position GPS, distance, and speed measurement; accelerometer: 100 Hz, 16 G Tri-Axial-Track impacts, accelerations, and decelerations as well as data source BL; Mag: 50 Hz, Tri-Axial; dimensions: 74 mm × 42 mm × 16 mm; SPI HPU based on Mining/Industrial Strength Electronics design; water resistance and data transmission: infra-red and weighs 56 g. Previous studies have shown that the GPS unit was tested for having a very high accuracy, demonstrated validity and inter-unit reliability, also, the intra-class correlation coefficients were high (>0.95) (Tessaro and Williams, 2018). Data collecting during training sessions and matches was performed in favorable weather and GPS satellite status.

### Data collection

Data collection was completed as in previous studies (Nobari et al., 2020b,d). At pre-session, we placed upright tracking units in the pouch of the manufacturer supplied belt, then the green light (GPS tracking) flashes were checked. At post-session, after verifying each unit was working properly, tracking units that collected the players' data were placed on the docking station. After 10 minutes, units turned off automatically, and data that were downloaded into docking memory were deleted from the units to prepare for the next session. The GPS units were tuned to the default SPI IQ Absolutes in this study. Duration in minutes and BL was calculated by accelerometer data, and it is designed to reflect both volume and intensity events of the accelerometer (acceleration). BL had replaced the original GPSports BL variable; it is an integrated loading variable used as a training load marker (BL) and work rate marker (BL/min), which we applied as a criterion for the training load in the current study. As to BL calculation, the following steps were repeated for each acceleration level: initialize the BL count to 0; magnitude of the acceleration vector (V) was calculated for the current acceleration (V = ax2 + ay2 + az2); normalize the magnitude vector (NV) by subtracting a national 1G (NV = V - 1.0 G); afterwards unscaled BL (USBL) was calculated through the formula USBLC = NV + [NV3]; then, the scaled BL (SBLC) was calculated taking into account the accelerometer logging rate (100 HZ) and exercise factor (EF) (SBLC = USBLC/100/EF); ultimately, final BL was calculated (BL = BL + SBLC).

### Calculation of workload parameters

#### Workload calculated

In this study, BL was used as the criterion for the training workload (Nobari et al., 2020b,c). Weekly training workload was used to calculate other workload parameters.

##### Acute workload calculation

We recorded the mean weekly AW of the team over the 48 weeks of the season.

##### Chronic workload calculation

We recorded the average weekly CW of the team between weeks 4 and 48 (45 weeks). The CW of each player was calculated with the following formula (Malone et al., 2017; Nobari et al., 2020a): where *n* = week number


$$CW=(AW_{n-1}+\;AW_{n-2}+AW_{n-3})\times \;0.333$$

##### ACWR calculation

The mean ratio between the AW and CW of the team was calculated and recorded with this variable. The ratio between the individual AW and CW was calculated with the following formula (Nobari et al., 2020a, d): where *n* = week number


(1) $$\text{ACWR}=\frac{AW_{n}}{(AW_{n-1}+\;AW_{n-2}+AW_{n-3})\;\times 0.333}$$

##### Δ-Acute load calculation

The AW variation between weeks was calculated through the formula: where *n* = week number


$$\begin{array}{l} \Delta -Acute=\frac{AWn}{CWn} \end{array}$$

##### AW level division

Difference between “high load” and “low load” weeks according to the average weekly AW of the team. “high load” was defined as AW ≥ 571 and “low load” was defined as AW <571. The cut-off point was established as follows: all the weeks of the season were ordered from highest to lowest AW load, the upper 1.3 was taken as high load and the lower 2.3 as low load.

##### CW level division

The difference between “high load” and “low load” weeks according to the average weekly CW of the team was calculated. “high load” was defined as CW ≥ 541 and “low load” was defined as CW <541. The cut-off point was established as follows: all the weeks of the season, with values of CW (45 last weeks) were ordered from highest to lowest CW load, the upper 1.3 was taken as high load and the lower 2.3 as low load.

##### ACWR level division

The difference between “high load” and “low load” weeks according to the average weekly ACWR of the team was calculated. “high load” was defined as ACWR ≥ 1.18 and “low load” was defined as ACWR <1.18. The cut-off point was established as follows: all the weeks of the season, with values of ACWR were ordered from highest to lowest ACWR load, the upper 1.3 was taken as high load and the lower 2.3 as low load.

##### Δ-Acute level division

The difference between “high load” and “low load” weeks according to the average weekly Δ-Acute of the team. “high load” was defined as Δ-Acute ≥ 1.19 and “low load” was defined as Δ-Acute <1.19. The cut-off point was established as follows: all the weeks of the season, with values of Δ-Acute, were ordered from highest to lowest Δ-Acute load, the upper 1.3 was taken as high load and the lower 2.3 as low load.

### Recording and calculation of injuries

Information on injuries was updated daily by the team's specialized medical staff. Based on a previous study, all injuries were recorded by type, location of the injury, and timing of the injury (Rogalski et al., 2013). The information used for the injuries is as follows:

#### The number of registered injuries

The total number of non-contact injuries per week for the team, over the 48 weeks of the season, was recorded with this variable.

#### Weekly injury

The existence or not of a non-contact injury in each of the 48 weeks of the season was recorded.

### Statistical analyses

The statistical software IBM Statistics 25 and R Studio 3.6.2. were used for statistical analyses. Accordingly, data were presented as means and standard deviations. A descriptive statistical analysis was realized, indicating the median values and interquartile range of the levels “high load” and “low load” for the variables “AW”, “CW”, “ACWR” and “Δ-AW,” as well as the total values. Non-parametric Mann-Whitney U tests were realized to compare the median of the load levels of the previous variables, checking the existence of statistically significant differences between them. A normality test, Kolmogorov-Smirnov, was performed, determining that the variables “number of injuries” and level of “AW”, “CW”, “ACWR” and “Δ-AW” did not follow a normal distribution. Additionally, a descriptive analysis of the number of injuries produced in the weeks of high and low load of each one of the variables was completed, as well as the calculation of the median of each one of them, both for the two levels of load as well as for the total. In the purpose of detecting statistically significant inter-group differences between the median of injuries of the “high load” and “low load” levels of the variables “high load” and “low load” for the variables “AW”, “CW”, “ACWR” and “Δ-AW”, non-parametric tests were carried out, taking into account, as factors, the load levels of each variable. A contrast of proportions was made to check the existence of significant differences between the levels of “high load” and “low load” of each variable and the weeks with the injury. To estimate the injury risk associated with high or low load level, the OR and RR were calculated. Finally, the variable “number of injuries” followed a Poisson distribution, so the Poisson test was performed to obtain lambda values (average number of injuries per week for each level of load), and the expected time until a new injury occurs. For checking possible significant differences between load levels, in addition to calculating the rate ratio, their confidence intervals 95% (CI 95%) were stated.

## Results

Overall, 12 non-contact injuries occurred during high load and 9 during low load weeks. The analysis revealed significant differences between “high” and “low” load levels for the AW, CW, ACWR, and Δ-AW variables (Table 1).

**Table 1:** Descriptive information of workload parameters based on high- and- low- level load.

**Workload variables**	**High load median (IQR)**	**Low load median (IQR)**	***P***	**Total median (IQR)**
AW (A.U.)	650 (608–732)	453 (395–492)	<0.001***	491 (409–611)
CW (A.U.)	577 (567–638)	473 (447–504)	<0.001***	501 (462–567)
ACWR (ratio)	1.30 (1.23–1.50)	0.91 (0.79–1.04)	<0.001***	1.06 (0.88–1.21)
Δ-AW (ratio)	1.35 (1.30–1.60)	0.83 (0.67–0.99)	<0.001***	0.99 (0.75–1.30)

IQR, Interquartile range; A.U., Arbitrary units; Acute workload (AW), High load (Weeks with loads ≥ 571 A.U.) and low load (Weeks with loads <571); Chronic workload (CW), High load (weeks with loads ≥ 541) and low load (weeks with loads <541); Acute chronic workload ratio (ACWR), High load (weeks with loads ≥ 1.18) and low load (weeks with loads <1.18); Increment in acute workload (Δ-AW), High load (Δ-AW ≥ 1.19) and low load (Δ-AW <1.19); p (p-value); ^***^(p <0.001).

It was appreciated that a superior mean of injuries occurred in the weeks of high load, in comparison to the weeks of low load for ACWR and Δ-AW, but no differences were observed in the variables AW and CW (Table 2).

**Table 2:** The relation between AW, CW, ACWR, and Δ-AW with non-contact injuries.

**Workload variables**	**High load**			**Low load**				**Total**		
	**Injuries**	**Weeks**	**M**	**Injuries**	**Weeks**	**M**	***p***	**Injuries**	**Weeks**	**M**
AW (A.U.)	10	16	0.63	11	32	0.34	0.193	21	48	0.44
CW (A.U.)	8	15	0.53	13	30	0.43	0.413	21	45	0.47
ACWR (ratio)	12	15	0.80	9	30	0.30	0.011**	21	45	0.47
Δ-AW (ratio)	12	16	0.75	9	31	0.29	<0.001***	21	47	0.45

Mean (M); Arbitrary units (A.U.); Acute workload (AW), High load (Weeks with loads ≥ 571 A.U.) and low load (Weeks with loads <571); Chronic workload (CW), High load (weeks with loads ≥ 541) and low load (weeks with loads <541); Acute chronic workload ratio (ACWR), High load (weeks with loads ≥ 1.18) and low load (weeks with loads <1.18); Increment in acute workload (Δ-AW), High load (Δ-AW ≥ 1.19) and low load (Δ-AW <1.19); p (p-value); ***(p <0.001); p (p-value); **(p <0.05) and *(p <0.05).

The contrast of proportions between the high and low load levels of each of the variables, and the number of weeks with and without injuries were calculated. Results indicated no significant differences between the proportions of weeks with and without injuries for the high and low load levels of the variables AC and CW. There were significant differences (*p* < 0.05) in the proportion of weeks without injuries between the high and low load weeks of the variables ACWR and Δ-AW.

In the four variables of interest, during the weeks with high-load levels, the OR of producing non-contact injuries were significantly higher for ACWR and Δ-AW in comparison with the weeks of low load. While in the RR, the significant differences were not found for all variables (Table 3).

**Table 3:** Injury risk related to different load levels and workload parameters with OR and RR.

**Workload variables**	**High load**		**Low load**		**OR**	**CI95%**		**RR**	**CI95%**	
	**INJURY (No injury)**	**TOTAL**	**INJURY (No injury)**	**TOTAL**		**MIN**	**MAX**		**MIN**	**MAX**
AW (A.U.)	8 (8)	16	11 (21)	32	1.91	0.56	6.48	1.31	0.76	2.28
CW (A.U.)	8 (7)	15	11 (19)	30	1.97	0.56	6.94	1.36	0.74	2.50
ACWR (ratio)	10 (5)	15	9 (21)	30	4.67	1.24	17.60	2.10	0.99	4.46
Δ-AW (ratio)	10 (6)	16	9 (22)	31	4.07	1.14	14.58	1.89	0.97	3.70

*Arbitrary units (A.U.); Acute workload (AW), High load (Weeks with loads ≥ 571 A.U.) and low load (Weeks with loads <571); Chronic workload (CW), High load (weeks with loads ≥ 541) and low load (weeks with loads <541); Acute chronic workload ratio (ACWR), High load (weeks with loads ≥ 1.18) and low load (weeks with loads <1.18); Increment in acute workload (Δ-AW), High load (Δ-AW ≥ 1.19) and low load (Δ-AW <1.19); Injury (weeks with injuries). No injury (weeks without injuries); Total (total weeks); OR (Odds Ratios); RR (Relative risk); CI95% (confidence interval); Min (Minimum); Max (Maximum)*.

Finally, it was observed that the time between injuries was shorter during weeks of high load compared to weeks of low load. However, only significant differences were found in the variables ACWR and Δ-AW between weeks of high and low load (Table 4).

**Table 4:** Relationship between injuries and different levels of load to find the expected time until new injuries.

**Workload variables**	**High load**		**Low load**		***p***	**Rate ratio**	**CI95%**	
	**λ**	**Expected time injury**	**λ**	**Expected time injury**			**MIN**	**MAX**
AW (A.U.)	0.63	1.6	0.34	2.94	0.17	1.82	0.69	4.72
CW (A.U.)	0.53	1.9	0.43	2.32	0.64	1.23	0.44	3.20
ACWR (ratio)	0.80	1.25	0.30	3.33	0.034*	2.66	1.03	7.17
Δ-AW (ratio)	0.75	1.33	0.29	3.45	0.036*	2.59	1.00	6.94

Arbitrary units (A.U.); Acute workload (AW), High load (Weeks with loads ≥ 571 A.U.) and low load (Weeks with loads <571); Chronic workload (CW), High load (weeks with loads ≥ 541) and low load (weeks with loads <541); Acute chronic workload ratio (ACWR), High load (weeks with loads ≥ 1.18) and low load (weeks with loads <1.18); Increment in acute workload (Δ-AW), High load (Δ-AW ≥ 1.19) and low load (Δ-AW <1.19); Expected time injury (Expected time between injuries. 1/λ); p (p-value); ^*^p <0.05; CI95% (Confidence interval); MIN (minimum); MAX (maximum); Rate ratio (Expected time injury low load/Expected time injury high load).

## Discussion

The purpose of this study was to investigate the association of workload with the OR and RR of non-contact injuries in male professional soccer players. Considering some studies (Jaspers et al., 2017; Malone et al., 2017) that investigated the relationship between workload and related injuries among professional soccer players, the monitoring of players' workload is important for injury prevention. In this study, external load monitoring was performed using GPS during each training and match session over the whole season. In this study, the number of recorded non-contact injuries across the soccer season amounted to 21, and it is similar in magnitude to that reported in a previous study (Ekstrand et al., 2011b). Also, according to Arazi et al., 21 subjects would be suitable for a relationship between workload parameters and injury rate with reduced chances of a Type II error (Arazi et al., 2020). For all participating soccer players, AW, CW, ACWR, and Δ-Acute workload parameters were calculated. Each variable while each variable was divided into two levels, “high-load” or “low-load”. Findings from this study support our research hypotheses. There were significant differences in the proportion of weeks with injury between high load and low load weeks. The OR of producing injuries without contact were significantly higher for ACWR and Δ-AW in comparison with the weeks of low load. Also, it was observed that the expected time between injuries was shorter between high load weeks rather than low load weeks. In sports science and medicine, rates are usually calculated using such atypical units as, training sessions, matches, player-season, etc.; (1) in this study, we tried to estimate the risk of non-contact injuries in 48 weeks of the season and weekly basis. Since risk is actionable information (2), it is normal to report the relative measures of risks, in regard to this, we reported relative risk (RR) and odds ratio (OR). Also, by reporting the predicted time between injuries, we tried to monitor the occurrence of injuries.

The relationship between the AW, CW, ACWR, and Δ-Acute with non-contact injuries in professional players was investigated. Statistically significant differences between “high” and “low” load levels were observed in all variables (AW, CW, ACWR, and Δ-Acute), and the mean of injuries for AW, ACWR, and Δ-AW variables was higher in the high load weeks in comparison to the low load weeks. In this regard, some workload-injury investigations showed similar relationships between absolute workloads and incidence of injury (Gabbett and Domrow, 2007; Killen et al., 2010). Also, a study in elite rugby players showed that the ones who had very high ACWR, as well as high CW, had the largest risk of injury (Hulin et al., 2016b), while another study stated that higher CW protects against injury (Cummins et al., 2013). Using ACWR provides a better understanding of the relationship between workload and injury risk in sports like soccer (Bowen et al., 2017), rugby (Hulin et al., 2016b), and cricket (Mcnamara et al., 2017). Gabbett et al. stated that the reason for using ACWR is that higher acute load compared to chronic load illustrates athlete unpreparedness, thus increasing injury risk (Gabbett et al., 2016), while Impellizzeri et al. concluded that no evidence shows the use of ACWR in managing training load to reduce injury risk (Impellizzeri et al., 2020a). Likewise, another study mentioned that using ACWR as an explanatory variable provides results which are always influenced by artifacts and artificial alterations. Artifact means any errors like reducing the variance of the explanatory variable, representation of any information, unjustified reclassifications, equipment, and technique. Considering that ACWR is a ratio and it is affected by its denominator, the players with low AW tend to have higher ACWR (Impellizzeri et al., 2020b). However, according to Malone et al., ACWR values between 1.00 and 1.25 A.U. were associated with lower injury incidence in professional soccer players (Malone et al., 2017), while ACWR values between 0.85 and 1.35 A.U. reduced the injury risk in rugby league groups (Hulin et al., 2016a). The estimation of injury risk associated with high load level compared to low load level was considered. In our study, the ACWRs were 1.30 (CI 95% = 1.23–1.50 A.U.) during high load level and 0.91 (CI 95%=0.79–1.04 A.U.) during low load level. Considering the previous findings, RR increased while participants were in high load levels while RR was reduced in low load levels.

High AW (Malone et al., 2017), cumulated weekly (Rogalski et al., 2013; Cross et al., 2016), and week to week changes (Cross et al., 2016) have been related to increased risk of injury, and recent studies declare that higher ACWR combined with low cumulative chronic workloads (Stares et al., 2018; Bowen et al., 2020), and rapid increases in workload (week-to-week changes) (Hulin et al., 2014; Bowen et al., 2020) may lead to a higher risk of injury. Colby et al. reported that there is a positive linear relationship between cumulative loads and injury risk (Colby et al., 2014), which is in agreement with our results. In our study, the mean of CW deviated from 0.53 to 0.43 in high load level to low load level, with a higher proportion of injuries in weeks of high load compared to weeks of low load, although there were no significant differences.

The OR describes the ratio of injury/disease odds given exposure status, although it is used especially in rare disease assumptions (Schmidt and Kohlmann, 2008). Therefore, if the rare disease assumption does not hold, it may be better to report RR. An OR and RR that are > 1.00 means that the risk is increased (Angelidis, 2020), which in the current study, OR and RR were > 1.00 for all variables. The OR and RR that are related to ACWR dedicated the largest number, 4.67 and 2.10, respectively, which determine that ACWR can better predict the risk of non-contact injuries. In this regard, Malone et al. suggested that ACWR between 1.0 and 1.25 is protective for players and results in reduced risk of injury (Malone et al., 2017), and in another study, Malone et al. demonstrated that high ACWR (>2.0) increase the risk of injury (Malone et al., 2016b). The current findings completely align with the Hulin et al. study that found GPS tracked ACWR predicted injury in elite rugby league players (Hulin et al., 2016b).

The relationship between injuries and different levels of load needed to find the expected time to new injuries was examined. In the current study, a Poisson test was done which provided two results. Firstly, the predicted time between injuries to new injury and secondly, lambda values. Lambda values (λ) indicate the average number of injuries per week related to both high load and low load levels. Regarding this value and ACWR, in high load level (weeks with loads ≥ 1.18 A.U.) the average number of injuries per week was 0.80, which was the largest number among all variables. The expected time between injuries which, interestingly, has an inverse relationship with λ was 1.33 for the Δ-AW variable, and 1.25 for the ACWR variable. Therefore, the predicted time between current injuries to new injuries is short and new injuries happen frequently. In line with this, recent studies showed that non-contact injuries had a high tendency for re-injury and several reports have been received even after returning to competition (Ekstrand et al., 2011a,b).

This study has some limitations. First, OR and RR are the same in rare events (Shrier and Steele, 2006), which means that by considering non-contact injuries in elite soccer players is not a rare event, it is more suitable to only report RR. The RR has been reported by its CI 95%, and it has been suggested that RR and CI 95% are presented for research investigating sports injuries (Barger, 2018). Second, the sample size in this study, although similar to previous studies (Murray et al., 2017b; Malone et al., 2019), was small. It is recommended that to be able to generalize the findings of this study to a broader population, the sample size should increase. Also, integrating both internal and external loads as metrics for injury risk should be considered in future studies (Malone et al., 2016a). While we are positive that players performed at their maximal intensity level during both training and competition, we do not have objectively measured data in support of this hypothesis. Furthermore, we did not control the hydration, dehydration, and cool-down and we recommend to researchers consider this in future studies. However, the strength of the present article is its specificity for male elite soccer players and highly accurate GPS units.

## Conclusions

In conclusion, in high load weeks compared to low load weeks, the number of injuries for AW, ACWR, Δ-AW variables were increased. Also, when considering ACWR, the injury risk during high-load levels was augmented. The predicted time to new injury decreased in high load weeks, therefore, the frequency of injuries increased. Our findings suggest that ACWR is an acceptable indicator for estimating injury risk and the time interval between injuries in soccer.

This study has practical applications for the monitoring of injury risk during high load weeks in professional soccer. It is recommended that coaches and practitioners regularly monitor the training load, ACWR, and AW. This monitoring may allow increased time between injuries, thus delaying new injuries in professional soccer players.

## Data availability statement

The original contributions presented in the study are included in the article/supplementary material, further inquiries can be directed to the corresponding authors.

## Ethics statement

This study was reviewed and approved by the Research Ethics Committee from the University of Mohaghegh Ardabili and University of Isfahan. The patients/participants provided their written informed consent to participate in this study.

## Author contributions

Conceptualization: HN, SK, AZ, and TB. Methodology: HN, AZ, UG, and TB. Data collection: HN. Analysis: HN, AZ, and UG. Writing—original draft preparation: HN, SK, and AZ. Writing—review and editing: HN, SK, UG, AZ, and TB. All authors contributed to the article and approved the submitted version.

## Funding

This work was funded by the Deutsche Forschungsgemeinschaft (DFG, German Research Foundation) – Projektnummer 491466077.

## Conflict of interest

The authors declare that the research was conducted in the absence of any commercial or financial relationships that could be construed as a potential conflict of interest.

## Publisher's note

All claims expressed in this article are solely those of the authors and do not necessarily represent those of their affiliated organizations, or those of the publisher, the editors and the reviewers. Any product that may be evaluated in this article, or claim that may be made by its manufacturer, is not guaranteed or endorsed by the publisher.

## Abbreviations

OR: odds ratio
RR: relative risk
AW: acute workload
CW: chronic workload
ACWR: acute to chronic workload ratio
Δ-Acute: AW variation
BL: body load
GPS: global positioning system
A.U.: arbitrary units
CI 95%: confidence interval
USBL: unscaled BL.
